# Lung Transplant Index: A Quality Improvement Initiative

**DOI:** 10.1097/pq9.0000000000000209

**Published:** 2019-09-19

**Authors:** Don Hayes, Bob Feeney, Donna J. O’Connor, Kerri L. Nicholson, Ashley E. Nance, Kelly K. Sakellaris, Nicole R. Dempster, Jaclyn D. Groh, Stephen E. Kirkby

**Affiliations:** From the * Department of Pediatrics, The Ohio State University College of Medicine, Columbus, Ohio; † Department of Internal Medicine, The Ohio State University College of Medicine Columbus, Ohio; ‡ Department of Surgery, The Ohio State University College of Medicine Columbus, Ohio; § Section of Pulmonary Medicine, Nationwide Children’s Hospital, Columbus, Ohio; ¶ Quality Improvement Services, Nationwide Children’s Hospital, Columbus, Ohio; ‖ Department of Psychiatry and Behavioral Health, Nationwide Children’s Hospital, Columbus, Ohio; ** The Heart Center, Nationwide Children’s Hospital, Columbus, Ohio.

## Abstract

Limited long-term survival is a recognized problem in adolescent/young adult lung transplant recipients. A quality improvement (QI) initiative included the development of a Lung Transplant Index (LTI) composed of key elements that we used as a comprehensive approach to screen and identify potential harms in this at-risk patient population.

**Methods:**

A single-center, uncontrolled QI study was completed from January 2014 to February 2019. The elements of the LTI are events that should have occurred within the most recent 12 months. If an element did not occur, it was counted as a missed element of preventing harm and summated later serving as the LTI score. Implementation of the LTI occurred on January 1, 2015, with a retrospective chart review of patients seen in clinic the prior year serving as baseline measures for comparison.

**Results:**

The year before implementing the LTI, numerous opportunities failed to identify preventable harm in our adolescent/young adult lung transplant population. The LTI resulted in a sustained reduction of these missed opportunities without negatively influencing patient/family satisfaction with lengthening of the clinic visit.

**Conclusions:**

A single-center QI initiative identified preventable harms in an adolescent/young adult lung transplant population and reduced the number of preventable harm elements not performed. Future work is needed to determine if this type of QI initiative is associated with less healthcare utilization.

## INTRODUCTION

With the introduction of the Preventable Harm Index in 2010, a standard was established at our institution to strive for a goal of zero preventable harm.^1^ With this new tool, our hospital had the means to track preventable elements which allowed for the implementation of quality improvement (QI) initiatives. With a higher Preventable Harm Index being undesirable, a lower value represents a better performance with fewer elements. Upon implementation of this index system, there was an immediate decrease in medical errors, improvement in patient safety and mortality, and initiation of cost savings at our hospital.^2–4^ Over time, experts at our institution have realized that the Preventable Harm Index is a component of a more comprehensive and encompassing Clinical Index, which is a longitudinal metric that totals the number of elements with the goal being a lower score compared with the previous period.^5^ Subsequently, individual programs at our hospital have instituted initiatives using a Clinical Index to evaluate and improve their performance.^5–8^

With adolescent lung transplant recipients having worse long-term survival compared with older adult patients,^9–11^ the mechanisms contributing to this age-related disparity are not completely understood. Barriers to adherence of treatment regimens and management recommendations are often a recognized problem in the adolescent patient population^12,13^ and may contribute to the survival differences in younger and older lung transplant recipients. Our multidisciplinary team believes that inadequate adherence is a component of an overarching deficiency of these patients and their families not understanding possible harms, for example not understanding the need to take medications at specific times to maintain immunosuppression or to avoid exposures to others with a viral infection. Therefore, we implemented an outpatient QI initiative using a Clinical Index to identify preventable harms in adolescent and young adult lung transplant recipients at our program that we termed the Lung Transplant Index (LTI). Our multidisciplinary team developed a comprehensive approach for screening through the LTI, to identify potential harms and to facilitate the education of each patient/family. The elements of the LTI are bundled and completed during clinic visits to achieve a score to identify preventable harm in our patient population. Our multidisciplinary team included 2 pediatric pulmonologists, 2 nurse coordinators, a QI coordinator, a dietician, a licensed psychologist, a licensed social worker, and an administrative associate.

## METHODS

**Fig. 1.**
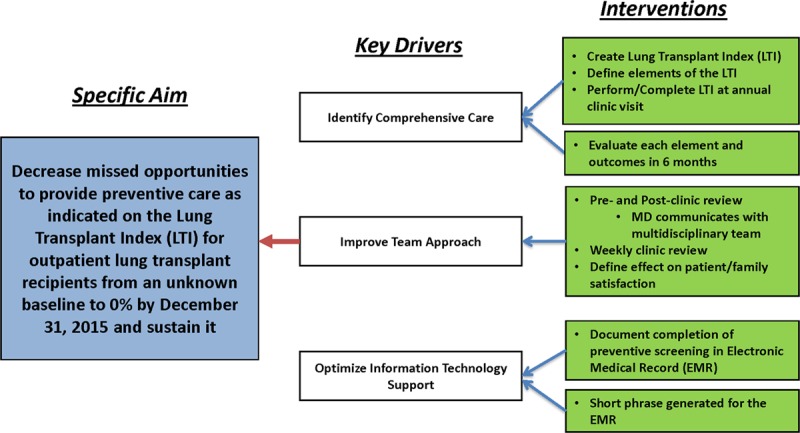
Key driver diagram for the LTI.

Using the Delphi method, our structured group, who were individual experts, completed 3 rounds of questionnaires that was facilitated by the lead author with revisions completed after each round. The final LTI was achieved upon the reaching of the consensus by our panel of experts. Figure 1 is the key driver diagram for our QI initiative with our specific aim being to decrease missed opportunities in providing preventive care through identifying preventable harm as indicated on LTI for outpatient lung transplant recipients from an unknown baseline to 0% by December 31, 2015, and sustain it for 3 years. Our key drivers were to identify comprehensive care, improve our team approach, and optimize information technology support to facilitate implementation and sustainment of our QI initiative. Our interventions were to create the LTI, define index elements, and perform/complete the LTI at each annual visit. Additional interventions were a pre- and post-clinic review by the treating pediatric pulmonologist the week before and after each clinic visit, communication between all members of the multidisciplinary team (listed above) through a weekly clinic review meeting, identify best processes in how to complete the LTI in an outpatient clinic setting, and assess the impact on patient/family satisfaction. Through the weekly clinic review model, all team members provided feedback about each patient and any concerns regarding harms identified with the LTI. Working with the information technology team, we developed a means to document the completion of the annual LTI for each patient in the electronic medical record to add this to the patient’s record.

Table 1 outlines each element of the LTI that our team felt was a source of possible harm in adolescent and young adult lung transplant recipients. The elements of the LTI included a pre- and post-test of medications including immunosuppression, antimicrobial prophylaxis, and other medications. Due to the importance of accurate medication documentation, the team felt that reconciliation was needed, so that was added to the LTI and was performed by the transplant coordinator at each clinic visit. Although medication reconciliation is a component of the National Patient Safety Goals, our team believed it should be included in the LTI to assess both prescription and nonprescription drugs due to the number of drug-drug interactions. Moreover, the medication reconciliation element was completed at each clinic visit but was only counted for 1 point for the LTI. Each patient undergoes annual laboratory and diagnostic testing as a means to screen for possible comorbidities or complications of lung transplantation, so these were considered separate elements with all testing under each representing 1 score for the LTI. Exposures of various types can cause significant morbidity and mortality in lung transplant recipients, so the LTI focused on specific ones for adolescent and young adult patients, including at school and while working. Preventive screening followed standard guidelines for patients of their age, which primarily focused on vaccinations, counseling for high-risk behaviors and trauma prevention, application of sunscreen during sun exposure, etc. Annual assessments by the multidisciplinary team are vital and were a separate element for the LTI. Finally, we felt that chronic complaints could often be overlooked and a sign of an underlying problem that may be preventable, so we included an assessment for chronic complaints/pain and sexual dysfunction. The LTI included these 11 elements that should have been assessed within the most recent 12 months. If an assessment did not occur, it is counted as a missed element and then added to the LTI with a later summation with each score being collected for each patient on an annual basis. A lower score was considered optimal and represented that our efforts to prevent harm for each patient was optimized. We generated a run chart as a means to assess the time series analysis.

**Table 1.**
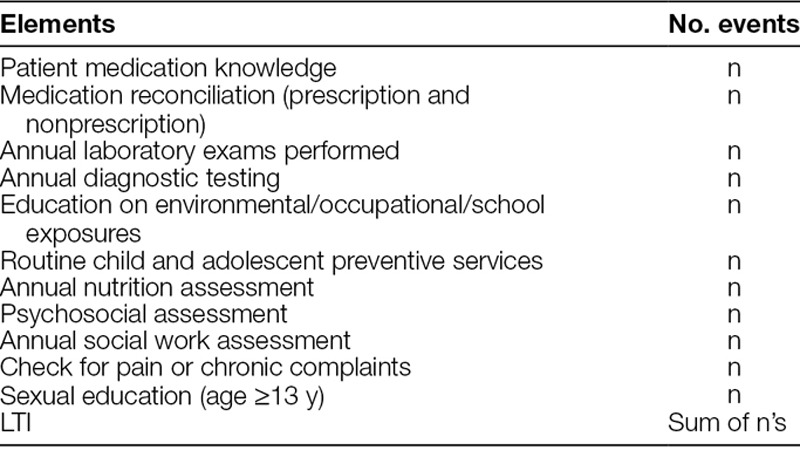
Lung Transplant Index Elements

To establish a baseline and for comparison, we retrospectively reviewed all clinic visits for the year before the implementation of the LTI and generated a score. With a start date of January 1, 2015, we reviewed the medical records for each patient included in our QI study from the year before (January 1, 2014, to December 31, 2014) and generated an LTI for that year to represent a baseline measurement for comparison.

With an intent to not significantly lengthen the clinic visit while completing both screening and education, a conscious effort to complete screening and education promptly was the goal. Although total clinic time was not collected, time to complete the entire LTI generally ranged between 4 and 5 minutes, with extension up to 10 minutes in some situations to elicit further important information if we detected harm(s).

## RESULTS

**Fig. 2.**
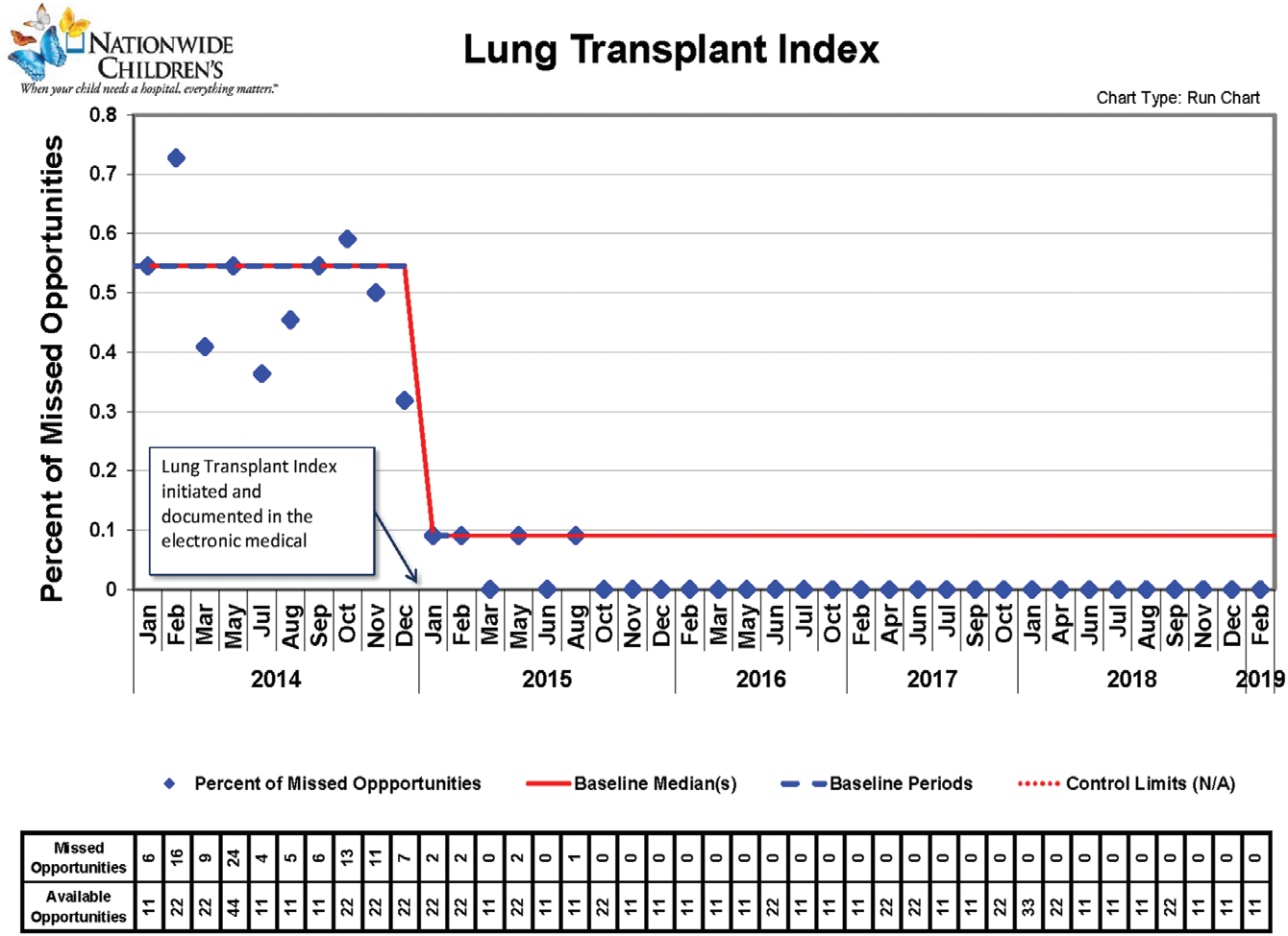
Run chart showing the percentage of missed opportunities for the LTI.

Using an uncontrolled before and after study design as described in the medical literature,^13^ we completed a QI study at our institution from January 2014 to February 2019. All patients followed at our program were included in the QI initiative (N = 14) with ages ranging from 13 to 36 years. Figure 2 is the run chart that shows the number of missed opportunities in the identification of preventable harm the year before and then after implementation of the LTI. Upon implementation, the LTI reduced the number of missed opportunities. This effect was sustainable for subsequent years. Although we did not specifically collect clinic times, patients and families believed that the LTI did not negatively influence their clinic experience based on our questioning of them after implementation.

## DISCUSSION

Our key finding is that a patient-centered QI initiative identified preventable harms in adolescent and young adult lung transplant recipients that were being missed. Before implementing the LTI, our approach to patient care was neglecting elements that placed our patient population at risk for harm. Modifying our approach resulted in optimized participation of the entire multidisciplinary team leading to a rapid improvement in the healthcare our team delivered.

We believe that avoiding possible harms in their home and their local community are vital for improving long-term outcomes of lung transplant recipients, especially the younger population. Important areas we addressed were improving patient understanding of their medications and confirming that all medications, both prescription and nonprescription, were being taken correctly. We also focused on timely completion of annual laboratory testing as well as annual diagnostic testing, such as pulmonary function testing and computed tomography imaging of the chest. Importantly, we focused on preventive measures around possible environmental, occupation, and school exposures. We often promoted the need to avoid others with signs and symptoms of a viral infection. From a multidisciplinary approach, we included the assessment of each team member in the LTI as each provider is needed to provide a holistic approach to the care of this patient population. For the chronic complaints, we focused more commonly on the nontransplant issues and chronic pain in this element. Finally, sexual education is rarely reported regarding this patient population, and our team felt it important to address this element on an annual basis. Based on the evidence from our QI initiative, we recommend further work to study the use of our Preventable Harm Index in lung transplant recipients as a means to reduce healthcare utilization for this patient population.

This QI study had several limitations, including a single-center design and small sample size. However, we successfully implemented a QI initiative that enhanced our identification of preventable harms in our patient population. Due to these limitations, we could not determine the true global clinical impact, so this area needs further research in larger cohorts to determine the relevance of our findings.

With minimal improvement in post-LTx survival for the past 2 decades, patient-centered care may serve as a potential facilitator of better long-term outcomes. Key factors that facilitated the success of our QI initiative were a commitment by a multidisciplinary team and support from an institution committed to improving quality of care.

In conclusion, the current analysis describes the success of an outpatient QI initiative that improved our screening process of preventable harms in adolescent and young adult lung transplant recipients. Currently, there is limited QI work published in solid organ transplantation. We believe that a comprehensive approach to QI initiatives across many centers would help define best practice, with regards to preventing harm and educating patients on health benefits in the younger lung transplant population. This type of collaboration may allow a platform to help ameliorate the contribution of preventable harm in long-term outcomes of adolescent and young adult lung transplant recipients.

## DISCLOSURE

The authors have no financial interest to declare in relation to the content of this article.
